# Nanostructured Electrospun Hybrid Graphene/Polyacrylonitrile Yarns

**DOI:** 10.3390/nano7100293

**Published:** 2017-09-25

**Authors:** Fahimeh Mehrpouya, Javad Foroughi, Sina Naficy, Joselito M. Razal, Minoo Naebe

**Affiliations:** 1 ARC Center of Excellence foe Electromaterials Science, Intelligent Polymer Research Institute, AIIM Facility, University of Wollongong, Wollongong, NSW 2119, Australia; fm392@uowmail.edu.au; 2 School of Chemical and Biomolecular Engineering, University of Sydney, Sydney, NSW 2006, Australia; sina.naficy@sydney.edu.au; 3 Institute for Frontier Materials, Deakin University, Geelong, VIC 3216, Australia; joselito.razal@deakin.edu.au (J.M.R.); minoo.naebe@deakin.edu.au (M.N.)

**Keywords:** liquid crystal graphene oxide, composite, nanofiber, electrospinning, twisted yarn

## Abstract

Novel nanostructured hybrid electrospun polyacrylonitrile (PAN) yarns with different graphene ratios were prepared using liquid crystal graphene oxide (LCGO) and PAN. It was found that the well-dispersed LCGO were oriented along the fiber axis in an electrified thin liquid jet during electrospinning. The graphene oxide sheets were well dispersed in the polar organic solvent, forming nematic liquid crystals upon increasing concentration. Twisted nanofibers were produced from aligned nanofibrous mats prepared by conventional electrospinning. It was found that the mechanical properties of the twisted nanofiber yarns increased even at very low LCGO loading. This research offers a new approach for the fabrication of continuous, strong, and uniform twisted nanofibers which could show promise in developing a novel carbon fiber precursor.

## 1. Introduction

Polyacrylonitrile (PAN) is a commercially-important polymer, mainly because it is the fiber precursor to about 90% of the carbon fiber manufactured today [1]. Carbon fibers exhibit high thermal stability, resistance to most solvents, high strength, and high stiffness. Notably, PAN-based carbon fibers are the preferred reinforcement material for structural composites where their superior strength and stiffness is combined with their light weight and low production cost compared to most metallic components [2]. Moreover, continuous advanced carbon nanofibrous yarns can be produced by carbonizing electrospun PAN-based yarns with improved mechanical properties [3,4]. These carbon nanofibers (CNFs) have been used in a wide variety of nanotechnology applications, including the development of materials and devices for energy storage, environmental, biomedical, electronics, and structural applications [3,5].

The degree of orientation of graphitic planes is the main parameter in determining the mechanical properties of CNFs. CNFs inherit their properties from the starting nanofibrous structure, and it has been reported that reinforced CNFs can be made from precursors with higher mechanical properties [3,6,7,8,9,10]. Hence, improving the properties of the PAN nanofibers (NFs) and their composites has been the subject of intensive investigations [1,11,12,13]. For instance, nanofillers such as montmorrilonite, carbon nanotubes, and/or graphene oxide (GO) have been added to PAN to make nanofibrous composites with improved mechanical strength, electrical conductivity, or thermal stability [14,15,16,17,18]. Although the previous studies demonstrated that carbon-based fillers at a loading of a few percent could enhance the mechanical properties of PAN nanofibers, the development of a perfectly structured precursor with optimum concentration of ingredients has been remained a challenge [19,20]. In this study, we present a novel preparation method of PAN/liquid crystalline GO (LCGO) composite nanofibrous twisted yarns which can be used as a precursor for carbon fiber production. Our results indicate the improved mechanical properties of the product with optimized concentration of ingredients. On the other hand, the introduced fabrication method of hybrid nanofibrous twisted yarns can be applied to develop functional materials from other 2D components such as transition metal dichalcogenides or 2D boron sheets (instead of GO) to improve different properties [21,22,23]. 

Graphene is a free-standing 2D crystal with one-atom thickness, and has become one of the most recent topics in the fields of materials science, physics, chemistry, and nanotechnology [24,25]. It has a large theoretical specific surface area (2630 m^2^ g^−1^), high intrinsic mobility (200,000 cm^2^ v^−1^ s^−1^), high Young’s modulus (~1.0 TPa), high thermal conductivity (~5000 Wm^−1^ K^−1^), and good electrical conductivity [26]. It is worth mentioning that in recent years, graphene fiber has become a new carbonaceous fiber with novel high mechanical and functional prospects [27].

Unlike pristine graphene which has limited processability, graphene oxide (GO) can be easily dispersed in many solvents due to the presence of various polar functional groups on its surfaces and edges [28,29,30]. In view of their excellent mechanical and physical properties, graphene and GO-based composites are expected to demonstrate enhanced properties compared to conventional composites [31]. The use of liquid crystalline GO dispersions enables the development of unique 3D assemblies with highly ordered macroscopic structures [4,31,32,33,34].

As mentioned above, this paper aims to create PAN/LCGO nanofibrous twisted composite yarns that can be used as a precursor for the fabrication of reinforced carbon fibers. These fibers could also be used for different applications in the fields of energy storage (e.g., supercapacitors) [35,36], bio-applications (e.g., enzyme immobilization) [1], and ultra-fast microfiltration of oil–water emulsion [37]. A highly-oriented molecular structure is needed for high-performance carbon fibers. Since a minor quantity of graphene sheets restrain the disorientation of the chain segments, this will cause an improvement in the molecular orientation of precursor fibers during spinning and stabilization [19]. For example, Chien et al. [38] fabricated composite carbon fibers with higher mechanical properties using continuous PAN/GO nanoribbon composite fibers as the precursor. Here, liquid crystal graphene oxide (LCGO) dispersed in an organic solvent was incorporated into PAN dissolved in the same solvent to prepare composite nanofibrous mats. These mats then were twisted to produce PAN/LCGO nanofibrous twisted composite yarns. The morphology as well as chemical and mechanical properties of the composites were studied by optical microscopy, field emission scanning electron microscopy (FESEM), transmission electron microscopy (TEM), Raman spectroscopy, differential scanning calorimetry (DSC), and mechanical properties testing.

## 2. Results

### 2.1. Rheological Behavior of LCGO and PAN/LCGO Composite Suspensions

**Figure 1:**
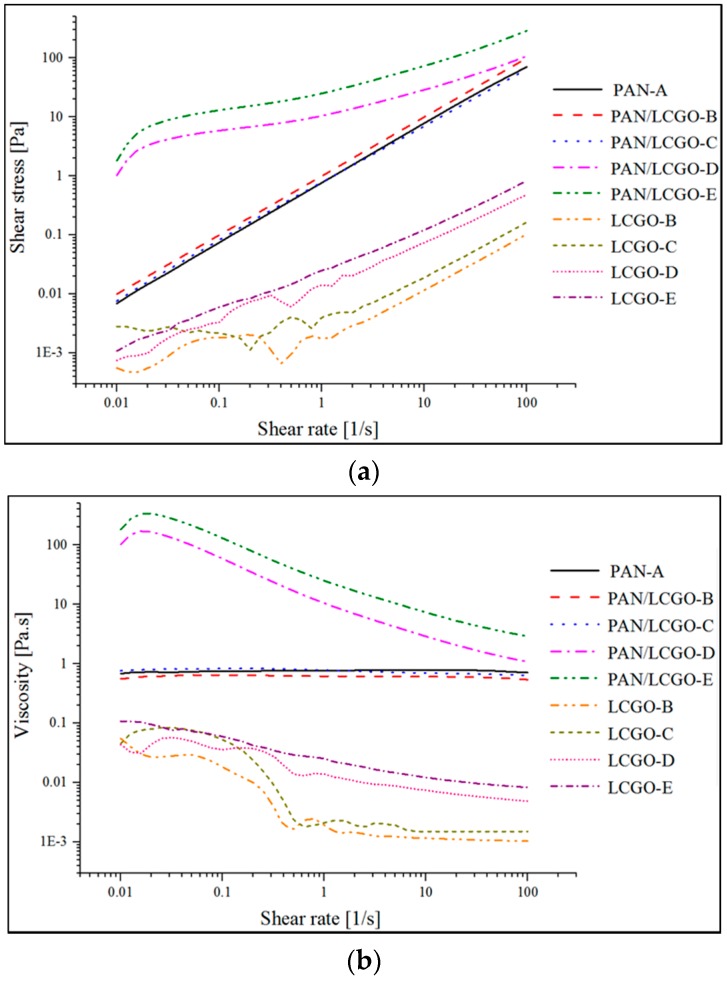
(**a**) Shear stress and (**b**) viscosity vs. shear rate curves of liquid crystal graphene oxide (LCGO) dispersions in *N*,*N*-dimethyl formamide (DMF) and polyacrylonitrile (PAN) and PAN/LCGO dispersions in DMF.

To evaluate the properties of PAN and hybrid PAN/LCGO solutions, rheological analysis of the spinning solutions were carried out. The rheological behavior of PAN, LCGO and PAN/LCGO dispersions are shown in Figure 1. Shear stress and viscosity of PAN solution and PAN/LCGO dispersions were characterized as the function of shear rate at 25 °C using the cone–plate configuration.

### 2.2. As-Prepared Hybrid Electrospun Nanofibers 

**Figure 2:**
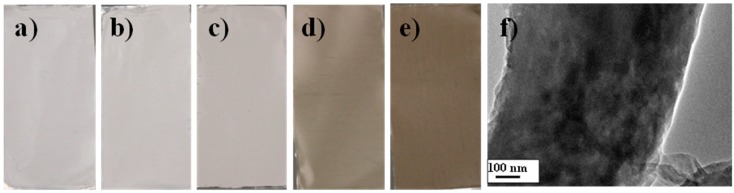
Photography of the as-prepared electrospun mate (**a**) PAN; (**b**) PAN/LCGO-B; (**c**) PAN/LCGO-C; (**d**) PAN/LCGO-D; and (**e**) PAN/LCGO-E; (**f**) TEM image of PAN/LCGO-D shows distribution of LCGO sheets through the nanofibrous structure.

The fabrication of PAN and hybrid PAN/LCGO nanofiber were carried out using an electrospinning machine. The 4 cm-wide ribbons consisting of aligned polymer nanofibers were formed on the drum collector (Figure 2a–e). Remarkably, with GO embedding, the color of electrospinning solutions and electrospun nanofibrous mats changed from white to dark brown, suggesting that the GO nanosheets were dispersed in the PAN substrate (Figure 2) [31,32]. In addition, the TEM images of PAN/LCGO spinning solutions were taken to evaluate the dispersion of LCGO in PAN as the polymeric matrix. The TEM image of PAN/LCGO-D hybrid nanofiber is presented in Figure 2f.

### 2.3. Morphology of PAN/LCGO Nanofibrous Mats and Twisted Yarns

**Figure 3:**
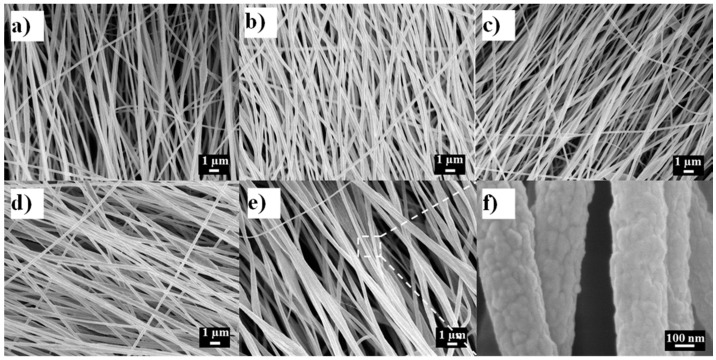
Field emission SEM (FESEM) images of as-spun nanofibers with different concentrations of LCGO, (**a**) PAN-A; (**b**) PAN/LCGO-B; (**c**) PAN/LCGO-C; (**d**) PAN/LCGO-D; (**e**) PAN/LCGO-E and (**f**) the high magnification FESEM image of PAN/LCGO-E to study the morphology of an individual nanofiber with highest concentration of LCGO.

Free-standing electrospun nanofibrous mats were successfully prepared from all PAN/LCGO concentrations presented in Table 1. The overall morphology of as-spun nanofibers (NF) is presented in Figure 3. As can be seen, the electrospun nanofibers had variable fiber diameters and structure, which was significantly affected by the addition of LCGO. As can be seen from Table 1, with the increase of LCGO loading, the average diameters of the composite nanofibers also increased.

Figure 3a–e also show that most of the nanofibers are oriented in one direction, which is in the direction of the rotation of the drum collector. As can be seen in Figure 3, the individual nanofibers with any concentrations of LCGO have an almost smooth structure. 

**Figure 4:**
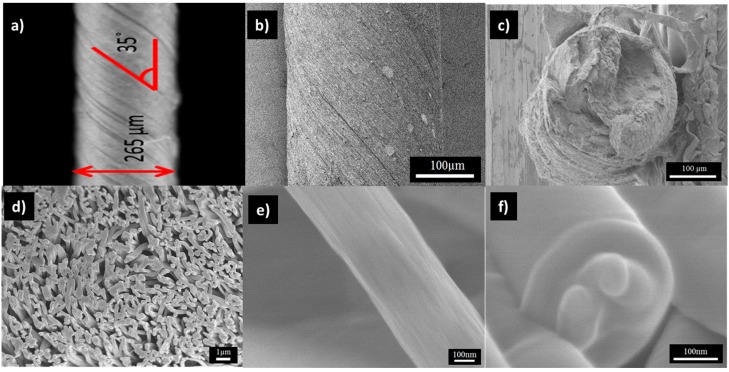
As-prepared electrospun nanofibres: (**a**) surface optical microscopy; (**b**–**d**) longitudinal and cross-sectional SEM images of the PAN/LCGO-D nanofibrous twisted yarn, respectively. (**e**,**f**) longitudinal and cross-sectional FESEM images of individual PAN/LCGO-D nanofiber, respectively.

Twisted yarns of electrospun nanofibers were obtained by twisting the electrospun mat. Figure 4a–c show the surface optical microscopy and longitudinal and cross-sectional SEM images of the PAN/LCGO-D nanofibrous twisted yarn. As can been seen from the surface morphology, the nanofibers were uniform, and predominantly oriented with a helix angle of ~35° to the yarn axis (Figure 4a,b). 

### 2.4. Raman Spectroscopy of PAN/LCGO Nanofibrous Twisted Yarns

Raman spectroscopy plays an important role in the structural characterization of graphitic materials [25]. A D-band in Raman spectra of pure PAN at 1320–1345 cm^−1^ corresponds to sp^3^ C–C bonds, indicating the disordered turbostratic structures, amorphous carbon, or defects in the curved graphene nanosheets. The G-band at 1580–1597 cm^−1^ corresponds to the in-plane tangential stretching mode of sp^2^ C–C bonds, expressing the ordered graphite crystallite structure and tangential shearing mode of the carbon atoms [18,26,27]. The relative intensity ratio of the D-band to the G-band, ID/IG—which is called the “*R*-value”—indicates the amount of quantitative characterization of the structurally ordered graphite crystallites in the carbonaceous materials. The width of the G band is also often used as an indicator of the level of graphitization (narrower G band indicates better graphitic structure) [27].

**Figure 5:**
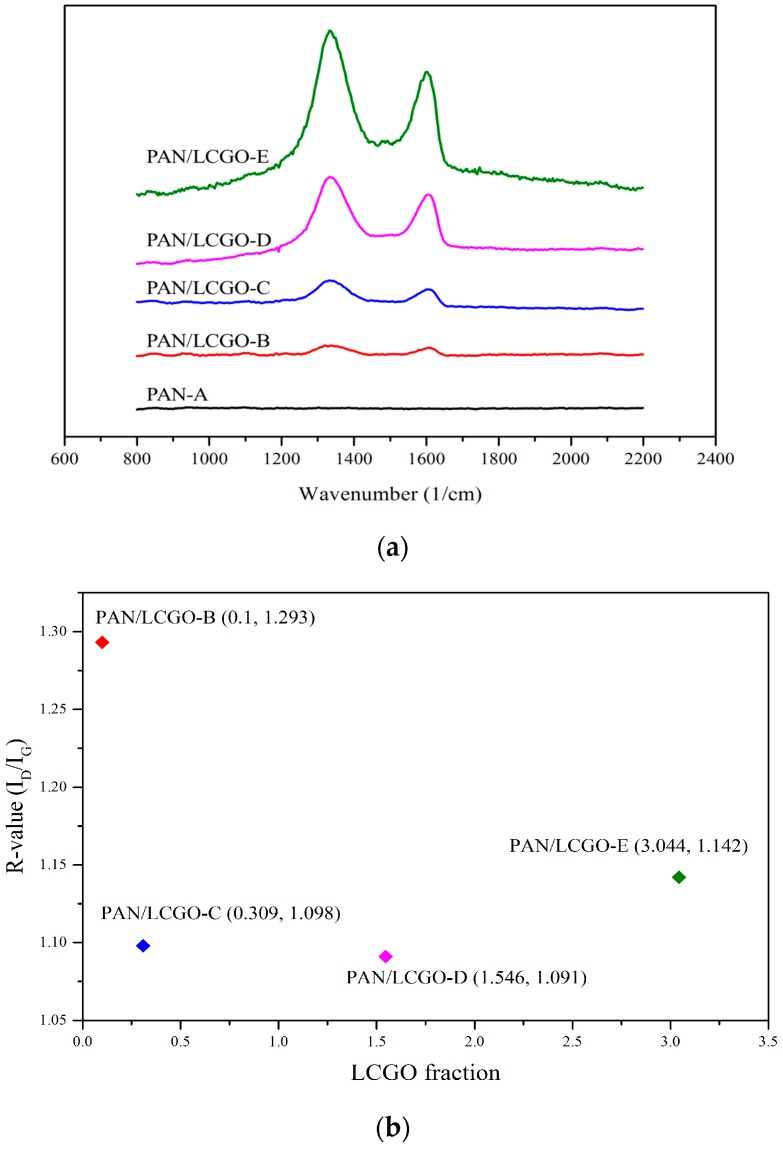
(**a**) Raman spectra of PAN/LCGO nanofibrous mats; and (**b**) *R*-values for the same samples.

Raman spectroscopy measurements in the region of 800–2200 cm^−1^ were performed to study the internal structure of the PAN/LCGO nanofibrous mats. The Raman spectra of pure PAN and LCGO/PAN nanofibrous samples (Figure 5a) show significant differences between the two materials. The *I*_D_/*I*_G_ ratio for the nanofibrous mats showed a peak at the 1.546 wt % LCGO fraction (sample PAN/LCGO-D), and this difference indicates that this sample has more ordered graphite crystallites (Figure 5b). Above 1.546 wt % LCGO fraction, the *R*-value increased (i.e., the crystallinity decreased) [15,36,39]. The samples with higher concentration of LCGO showed improved graphitic structure, as indicated by smaller *R* (Figure 5b). These results suggest that the significant improvements in the graphitic structure of the resulting CNFs were a direct consequence of the addition of a small amount of LCGO into the electrospinning solutions.

### 2.5. Differential Scanning Calorimetry Thermograms of PAN/LCGO Nanofibrous Twisted Yarns 

**Figure 6:**
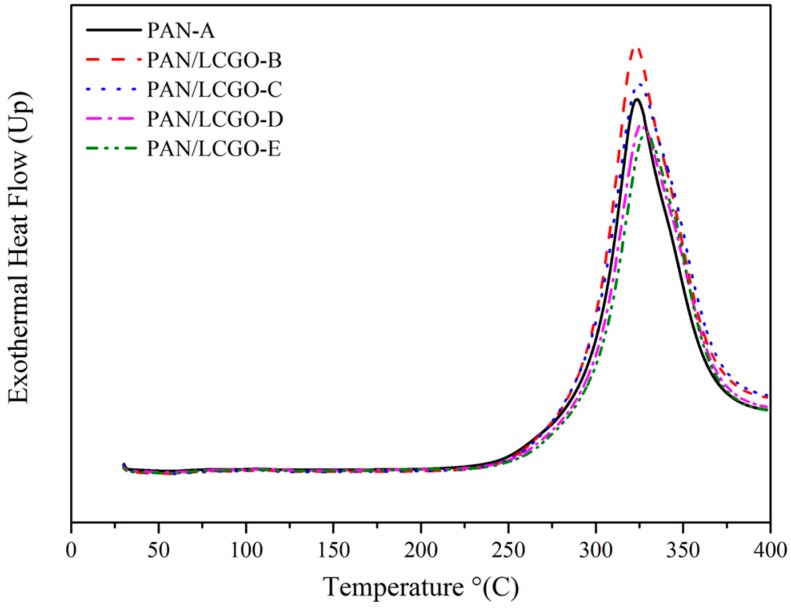
Differential scanning calorimetry (DSC) curves of the PAN and PAN/LCGO nanofibrous twisted yarns.

The effect of LCGO addition on the thermal properties of electrospun PAN/LCGO composite nanofibrous twisted yarns was investigated using DSC. For comparison, the DSC exothermic curve of PAN nanofibrous mat is also illustrated in Figure 6. The stabilization temperature and heat of fusion (Δ*H*) for blends are demonstrated in Table 2. The exothermic peak for pure PAN is 324 °C. Addition of only 0.3 wt % LCGO to the PAN solution led to an increase in stabilization temperature to 329 °C for the resultant mat. In general, the stabilization temperature increased because of LCGO addition. At the same time, a significant decrease in the heat of fusion (from 5036 to 4366 J/g) was observed with inclusion of LCGO in electrospun nanofibers.

### 2.6. Mechanical Properties of PAN/LCGO Nanofibrous Twisted Yarns 

**Figure 7:**
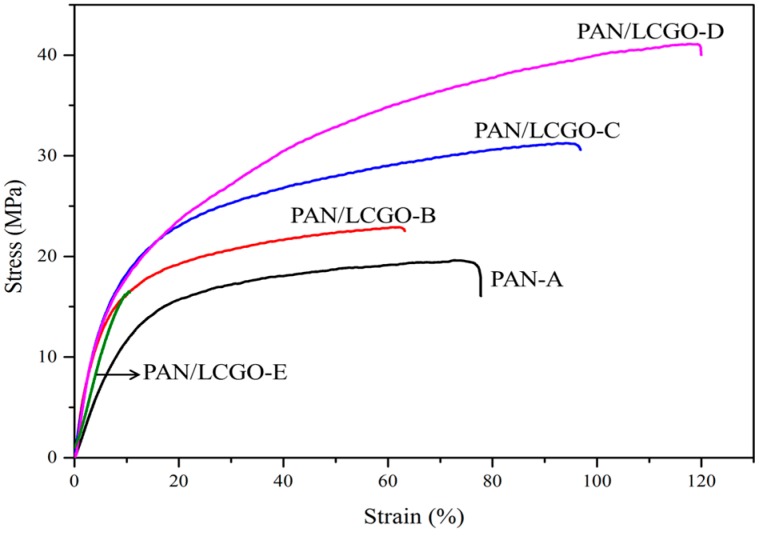
Stress vs. strain curves for as-prepared electrospun twisted PAN and PAN/LCGO yarns.

The mechanical properties of twisted electrospun PAN and hybrid PAN/LCGO yarns are shown in Figure 7. Stress–strain curves obtained from each of the twisted PAN and hybrid PAN/LCGO yarns showed a significant difference in mechanical properties. Analysis of these curves indicates a stress at break of 41.1 MPa with 119.1% strain for the as-prepared twisted PAN/LCGO-D yarn, compared with 19.6 MPa stress with 75.53% strain for the as-prepared twisted PAN-A yarn. The Young’s modulus of these yarns was 366.42 MPa and 145.35 MPa for the hybrid twisted PAN/LCGO-D and PAN-A yarns, respectively. The average values of tensile modulus and ultimate tensile strength of the as-prepared PAN/LCGO nanofibrous twisted yarns are summarized in Table 3.

## 3. Discussion

It can be seen in Figure 1 that shear stress increased with shear rate while viscosity decreased. Moreover, while PAN solution and dilute PAN/LCGO dispersions exhibited Newtonian behavior, LCGO dispersions at higher concentration displayed a shear thinning trend. The highly-concentrated PAN/LCGO dispersions had considerably higher viscosity and shear stress at low shear rates compared to PAN solution and dilute PAN/LCGO dispersions (more than 100 times). At higher shear rates, however, the viscosity continuously decreased with shear rate, converging to that of PAN and dilute PAN/LCGO. Similarly, the shear rate of PAN solution and dilute PAN/LCGO dispersions increased linearly with shear rate, while more concentrated PAN/LCGO dispersions behaved differently. After a sharp increase in the shear stress of concentrated PAN/LCGO dispersions with increasing shear rate, shear stress continued to increase linearly as a function of shear rate and again followed the dilute PAN/LCGO dispersions pattern at high shear rates. The observed shear thinning in rheological behavior of concentrated PAN/LCGO can be attributed to the alignment of LCGO plates in the dispersion at higher shear rates. Additionally, the change in the slope of shear stress as a function of shear rate at low shear rate values in concentrated PAN/LCGO dispersions agrees with this observation.

On the other hand, the trend in the average diameter of nanofibers (Figure 3) can be explained by the fact that higher concentration of LCGO resulted in more viscous PAN/LCGO dispersions, and hence affected the drawing of filaments during the whipping motion of filaments during the electrospinning process, resulting in thicker nanofibers [11]. Apart from viscosity, other material variables such as electrical conductivity and surface tension of the electrospinning solutions were impacted by the LCGO loading. All these parameters are influential variables in defining the final morphology of the electrospun fibers [11].

The electrospinning parameters were optimized to obtain uniform nanofiber structures, as is evident from Figure 3. However, as can be observed in Figure 3, the sample PAN/LCGO-E with highest concentration of LCGO displayed rough and thicker regions which suggest it contained large graphene oxide sheets. It is known that GO sheets bend and fold easily into various shapes contingent upon substrate or temperature [3]. It is worth mentioning that a small amount of LCGO can remarkably change the solution properties of PAN. Because LCGO disperses well on DMF, the composite polymer solution, with higher concentrations of LCGO, can be divided into GO-rich domain and GO-scarce domain, which may lead to instability of the liquid jet during the electrospinning process. So, the beaded structures were formed.

Similar to conventional textile yarns, twisting is a useful fiber processing technique to improve yarn strength. Clearly, untwisted yarn displayed poor mechanical properties, and usually mechanical properties of twisted yarns are improved with the increase of twist level [40]. As-prepared electrospun PAN and hybrid PAN/LCGO nanofibers were transformed into yarn by twisting. The sample with higher ratio of the graphene (PAN/LCGO-E) showed lower mechanical properties compared to other samples (see Table 3). This phenomenon could be explained by aggregation of graphene in the polymer matrix due to a higher amount of LCGO in spinning solution. Consequently, as-spun PAN/LCGO-E nanofibers showed an enormously non-uniform structure and could not be twisted uniformly into the yarn. 

Considering the DSC thermograms of the twisted yarns, a clear increase in stabilization temperature and a corresponding decrease in Δ*H* could be realized with inclusion of LCGO [41,42]. The increase in the stabilization temperature of PAN/LCGO fibers upon the addition of LCGO content as well as the decrease in the Δ*H* could be due to the intermolecular interactions between PAN and LCGO, particularly when being heat treated during thermal analysis. This intermolecular interaction justifies the improvement in graphitic structure shown by Raman analysis as a result of LCGO inclusion. These findings are significant for the development of high-performance carbon nanofiber structures using nano-enhanced precursor materials.

The addition of LCGO increased yarns’ Young’s modulus and their strength. Yield points (maximum in the peak stress–strain curve) associated with a deformation mechanism could be detected for the different composites. From the slope of the inelastic part of the stress–strain curve, increasing the concentration of LCGO in samples improved the mechanical properties of PAN/LCGO nanofibrous twisted yarns. As it was previously shown, using fibers with improved mechanical properties as precursors for making carbon fibers can result in carbon fibers with improved properties [4]. The low twisting saturation number for PAN/LCGO-E due to its high average diameter and diameter heterogeneity resulted in the deviation from the trend for mechanical properties. The comparison of the result of Raman spectroscopy and stress–strain plots show that increasing the concentration of LCGO in electrospinning solutions can improve mechanical properties by improving the graphitic structure. 

## 4. Materials and Methods 

LCGO dispersion in *N*,*N*-dimethyl formamide (DMF) was synthesized in our laboratory, following a method presented elsewhere [34,43]. The PAN (average molecular weight ~150,000 g mol^−1^) powder was obtained from Sigma-Aldrich (Sydney, Australia) and used without further purification. DMF was used as received (Sigma-Aldrich).

Known amounts of LCGO in DMF were added to PAN solutions (according to Table 1) and mixed by magnetic stirring for 48 h to prepare the electrospinning dope solutions. The mass ratio of the PAN was 10 wt % in all solutions. The electrospinning set-up consisted of a syringe which injected the electrospinning solution at the rate of 1 mL h^−1^ onto a drum collector rotating at 2000 rpm. The distance and voltage between the syringe tip and the collector was maintained at 15 cm and 15 kV, respectively. Electrospinning was carried out at ambient temperature inside a humidity-controlled chamber with humidity between 30% and 40%. 

The nanofiber ribbons with average initial length of 57 cm were easily removed from the collector to be twisted into yarns. For this purpose, each ribbon was first immersed in ethanol and then connected to two motors’ shafts which rotated at 42 rpm for 17 min; thus, the ribbons were twisted into yarns. It is worth mentioning that further increase in twist resulted in breakage of the yarns [32]. Table 1 shows the percentage of LCGO in NF mats, which was calculated as:

(1) $$\mathrm{GO}\;\left(\mathrm{in}\;\mathrm{NFs}\;\mathrm{mats}\right)=\frac{m\left(\mathrm{GO}\right)}{m\left(\mathrm{GO}\right)+m\left(\mathrm{PAN}\right)}\times 100$$

## 5. Conclusions

The incorporation of a very small amount of liquid crystal graphene oxide into PAN matrix has been shown to have significant effects on the graphitic structure and preferred orientation of the composite nanofibrous twisted yarns formed from these dispersions. Consequently, electrospun PAN yarn and PAN/LCGO nanocomposite yarns have been prepared. It was found that the mechanical properties of as-prepared PAN/LCGO yarn have significantly enhanced compared to the electrospun PAN yarn.

The results revealed that the incorporation of LCGO effectively enhanced the mechanical properties of the composite nanofibers. It is expected that far better properties can be attained from the carbon yarns which will be made from these precursors, but still more studies are needed to further understand and manipulate the effect of using LCGO in electrospinning dispersions to produce advanced carbon yarns.

## Figures and Tables

**Table 1:** Electrospinning solutions and final percentage of GO in nanofibrous (NF) mats.

Sample	Solvent	PAN (wt %)	LCGO (wt %)	GO (in NFs Mats) %	Average NFs Diameter (nm)
PAN-A	DMF	10	----	---	252
PAN/LCGO-B	DMF	10	0.01	0.099	251
PAN/LCGO-C	DMF	10	0.031	0.309	311
PAN/LCGO-D	DMF	10	0.157	1.546	345
PAN/LCGO-E	DMF	10	0.314	3.044	490

**Table 2:** The stabilization temperature and heat of fusion (Δ*H*) of PAN and PAN/LCGO nanofibrous twisted yarns.

Sample	DSC Max Peak (°C)	Δ*H* (J/g)
PAN-A	324	5036
PAN/LCGO-B	323	4827
PAN/LCGO-C	325	4660
PAN/LCGO-D	327	4818
PAN/LCGO-E	329	4366

**Table 3:** Mechanical properties of PAN/LCGO nanofibrous twisted yarns at Yield points.

Sample	PAN-A	PAN/LCGO-B	PAN/LCGO-C	PAN/LCGO-D	PAN/LCGO-E
Young’s Modulus (MPa)	145.35	312.5	332.45	366.42	233.56
Stress (MPa)	19.60	22.90	31.20	41.10	16.5
Strain (%)	75.53	62.32	93.40	119.10	13.15
